# Twenty-Year Change in Severity and Outcome of Ischemic and Hemorrhagic Strokes

**DOI:** 10.1001/jamaneurol.2021.4346

**Published:** 2021-12-06

**Authors:** Kazunori Toyoda, Sohei Yoshimura, Michikazu Nakai, Masatoshi Koga, Yusuke Sasahara, Kazutaka Sonoda, Kenji Kamiyama, Yukako Yazawa, Sanami Kawada, Masahiro Sasaki, Tadashi Terasaki, Kaori Miwa, Junpei Koge, Akiko Ishigami, Shinichi Wada, Yoshitaka Iwanaga, Yoshihiro Miyamoto, Kazuo Minematsu, Shotai Kobayashi

**Affiliations:** 1 Department of Cerebrovascular Medicine, National Cerebral and Cardiovascular Center, Suita, Japan; 2 Department of Medical and Health Information Management, National Cerebral and Cardiovascular Center, Suita, Japan; 3 Department of Neurology, Saiseikai Fukuoka General Hospital, Fukuoka, Japan; 4 Department of Neurosurgery, Nakamura Memorial Hospital, Sapporo, Japan; 5 Department of Stroke Neurology, Kohnan Hospital, Sendai, Japan; 6 Stroke Center, Okayama Kyokuto Hospital, Okayama, Japan; 7 Department of Stroke Science, Akita Cerebrospinal and Cardiovascular Center, Akita, Japan; 8 Department of Neurology, Japanese Red Cross Kumamoto Hospital, Kumamoto, Japan; 9 Medical Corporation ISEIKAI, Osaka, Japan; 10 Shimane University School of Medicine, Izumo, Shimane, Japan

## Abstract

**Importance:**

Whether recent changes in demographic characteristics and therapeutic technologies have altered stroke outcomes remains unknown.

**Objective:**

To determine secular changes in initial neurological severity and short-term functional outcomes of patients with acute stroke by sex using a large population.

**Design, Setting, and Participants:**

This nationwide, hospital-based, multicenter, prospective registry cohort study used the Japan Stroke Data Bank and included patients who developed acute stroke from January 2000 through December 2019. Patients with stroke, including ischemic and hemorrhagic strokes, who registered within 7 days after symptom onset were studied. Modified Rankin Scale scores were assessed at hospital discharge for all patients.

**Exposure:**

Time.

**Main Outcomes and Measures:**

Initial severity was assessed by the National Institutes of Health Stroke Scale for ischemic stroke and intracerebral hemorrhage and by the World Federation of Neurological Surgeons grading for subarachnoid hemorrhage. Outcomes were judged as favorable if the modified Rankin Scale score was 0 to 2 and unfavorable if 5 to 6.

**Results:**

Of 183 080 patients, 135 266 (53 800 women [39.8%]; median [IQR] age, 74 [66-82] years) developed ischemic stroke, 36 014 (15 365 women [42.7%]; median [IQR] age, 70 [59-79] years) developed intracerebral hemorrhage, and 11 800 (7924 women [67.2%]; median [IQR] age, 64 [53-75] years) developed subarachnoid hemorrhage. In all 3 stroke types, median ages at onset increased, and the National Institutes of Health Stroke Scale and World Federation of Neurological Surgeons scores decreased throughout the 20-year period on multivariable analysis. In ischemic stroke, the proportion of favorable outcomes showed an increase over time after age adjustment (odds ratio [OR], 1.020; 95% CI, 1.015-1.024 for women vs OR, 1.015; 95% CI, 1.011-1.018 for men) but then stagnated, or even decreased in men, on multivariate adjustment including reperfusion therapy (OR, 0.997; 95% CI, 0.991-1.003 for women vs OR, 0.990; 95% CI, 0.985-0.994 for men). Unfavorable outcomes and in-hospital deaths decreased in both sexes. In intracerebral hemorrhage, favorable outcomes decreased in both sexes, and unfavorable outcomes and deaths decreased only in women. In subarachnoid hemorrhage, the proportion of favorable outcomes was unchanged, and that of unfavorable outcomes and deaths decreased in both sexes.

**Conclusions and Relevance:**

In this study, functional outcomes improved in patients with ischemic stroke during the past 20 years in both sexes presumably partly owing to the development of acute reperfusion therapy. The outcomes of patients with hemorrhagic stroke did not clearly improve in the same period.

## Key Points

**Question:**

Did the initial neurological severity and functional outcomes of patients with stroke change throughout a 20-year period?

**Findings:**

In this hospital-based, multicenter, prospective registry involving 183 080 patients with acute stroke, initial neurological severity showed a decrease over time in all stroke types. Functional outcome at hospital discharge improved in patients with ischemic stroke but no longer showed improvement after adjustment by reperfusion therapy and others; it did not clearly improve in patients with hemorrhagic stroke.

**Meaning:**

Twenty-year changes in functional outcomes after ischemic and hemorrhagic strokes showed different trends presumably partly owing to differences in the development of acute therapeutic strategies.

## Teaser

This cohort study assesses secular changes in initial neurological severity and short-term functional outcomes of patients with acute stroke by sex using a large population.

## Introduction

Several population-based registries have demonstrated long-term changes in age at onset, incidence, and mortality of stroke; the trends differed by changes in lifestyle and medical conditions.^1,2,3,4,5,6^ In Japan, both the age-adjusted incidence and mortality of stroke decreased drastically during the past half century, but these trends slowed in resent decades.^2^ In addition, notable technologies were introduced domestically every 5 years, with official approval for intravenous thrombolysis in 2005, official approval for the first device for mechanical thrombectomy in 2010, and far-reaching spread of stent retrievers for thrombectomy in 2015.^7^ However, whether the changes in demographic characteristics and therapeutic technologies altered stroke severity and functional outcomes from a long-range perspective remains unknown. Such changes could be clarified by long-term hospital-based registries.

Twenty-six countries have been reported to have nationwide standardized data sets for acute stroke.^8^ Some of these reported long-term changes in stroke severity; the Austrian Stroke Unit Registry, involving 53 126 patients with intracerebral hemorrhage (ICH) between 2008 and 2016,^9^ and the National Acute Stroke Israeli registry, involving 6693 patients with ischemic stroke (IS) and ICH between 2004 and 2013,^10^ showed a decrease in severity assessed by the National Institutes of Health Stroke Scale (NIHSS). Functional outcome assessed by the modified Rankin Scale (mRS) and other scales is an important indicator for most nationwide data sets,^11,12^ but its long-term changes were minimally discussed.

The Japan Stroke Data Bank (JSDB), previously the Japan Standard Stroke Registry Study, is a 20-year-long nationwide hospital-based registry in which the NIHSS scores on emergent hospital admission and the mRS scores at hospital discharge have been required fields from the beginning.^13,14,15,16,17^ Higher scores for both scales in female patients with IS than their male counterparts on multivariable analysis were reported from the registry.^14^ Other hospital-based registries reported similar sex-related differences.^18,19,20,21,22,23,24,25,26,27^ In addition to older onset age in women than men, the tendency for social isolation, poorer premorbid activity, limited access to medical resources, frequent prestroke and poststroke depression, and several more reasons could cause poor stroke conditions for women.^28,29,30^ Thus, we hypothesized that long-term changes in severity and outcome are also affected by sex. In the present study, secular changes in initial neurological severity and short-term outcomes of patients with stroke assessed using NIHSS and mRS scores were determined based on a large hospital-based patient population by sex.

## Methods

### Study Design and Setting

The JSDB study is an ongoing, hospital-based, multicenter, prospective registry of hospitalized patients with acute stroke or transient ischemic attack based on a web database from 130 academic or regional stroke centers distributed evenly throughout Japan (eTable 1 in Supplement 1). The unique aspects of this nationwide registry include standardized clinical information, detailed diagnosis, and acute management by stroke specialists. Patients’ data were prospectively recorded by the study physicians or clinical report coordinators in each institute using a standardized database form online. This study followed the Strengthening the Reporting of Observational Studies in Epidemiology (STROBE) reporting guidelines. The study protocol was approved by the institutional ethical board at National Cerebral and Cardiovascular Center. Individual consent for entry into the database was waived, with an opt-out consent method used instead. The data set of the JSDB study is open to the investigators who participate in this registry.

### Participants

For the present analyses, patients with any stroke who were registered within 7 days after symptom onset were eligible for inclusion. Clinical information on demographics, stroke types, and IS subtypes according to the criteria of the Trial of Org 10172 in Acute Stroke Treatment,^31^ history of any stroke, and performance of acute reperfusion therapy (ie, intravenous thrombolysis or acute endovascular therapy mainly by mechanical thrombectomy) were collected from the database.

The outcome measures were (1) neurological severity at the emergent visit corresponding with the NIHSS score for patients with IS or ICH and the World Federation of Neurological Surgeons (WFNS) grading for those with subarachnoid hemorrhage (SAH); (2) the proportion of patients with favorable functional outcomes at hospital discharge corresponding with an mRS score of 0 to 2; (3) the proportion of patients with unfavorable functional outcomes at hospital discharge corresponding with an mRS score of 5 to 6; and (4) in-hospital mortality.

### Statistical Analysis

Continuous data are reported as median and IQR and categorical data are presented as numbers and percentages. Box plots for age and NIHSS score (WFNS for SAH) were conducted for 4 categories of years (2000-2005, 2006-2010, 2011-2015, and 2016-2019) by sex. Mann-Whitney *U* and χ^2^ tests were used to test the significance of differences between 2 groups for continuous and categorical variables, respectively. To determine secular changes in the outcomes or changes in the outcomes of patients with IS by the above 4 categories, multilevel mixed-effect multivariable regression and logistic regression using the institutes as random intercepts were performed, adjusted for sex, age, NIHSS score (WFNS for SAH), history of stroke, and reperfusion therapy. The Cochran-Armitage test was also performed to check for year trends. Significance was defined as a *P* value less than .05. Statistical analyses were performed with STATA version 16 (StataCorp).

## Results

Of 214 924 patients registered in the JSDB between January 2000 and December 2019, 15 044 had a diagnosis of transient ischemic attack, 10 412 registered 8 or more days after onset, and 6388 had unavailable data on demography or stroke types and were excluded from the study (eFigure in Supplement 1). Of the remaining 183 080 patients (77 089 women [42.1%]; median [IQR] age, 73 [64-81] years) included in the study, 135 266 developed IS (53 800 women [39.8%]; median [IQR] age, 74 [66-82] years), 36 014 developed ICH (15 365 women [42.7%]; median [IQR] age, 70 [59-79] years), and 11 800 developed SAH (7924 women [67.2%]; median [IQR] age, 64 [53-75] years).

**Figure 1.**
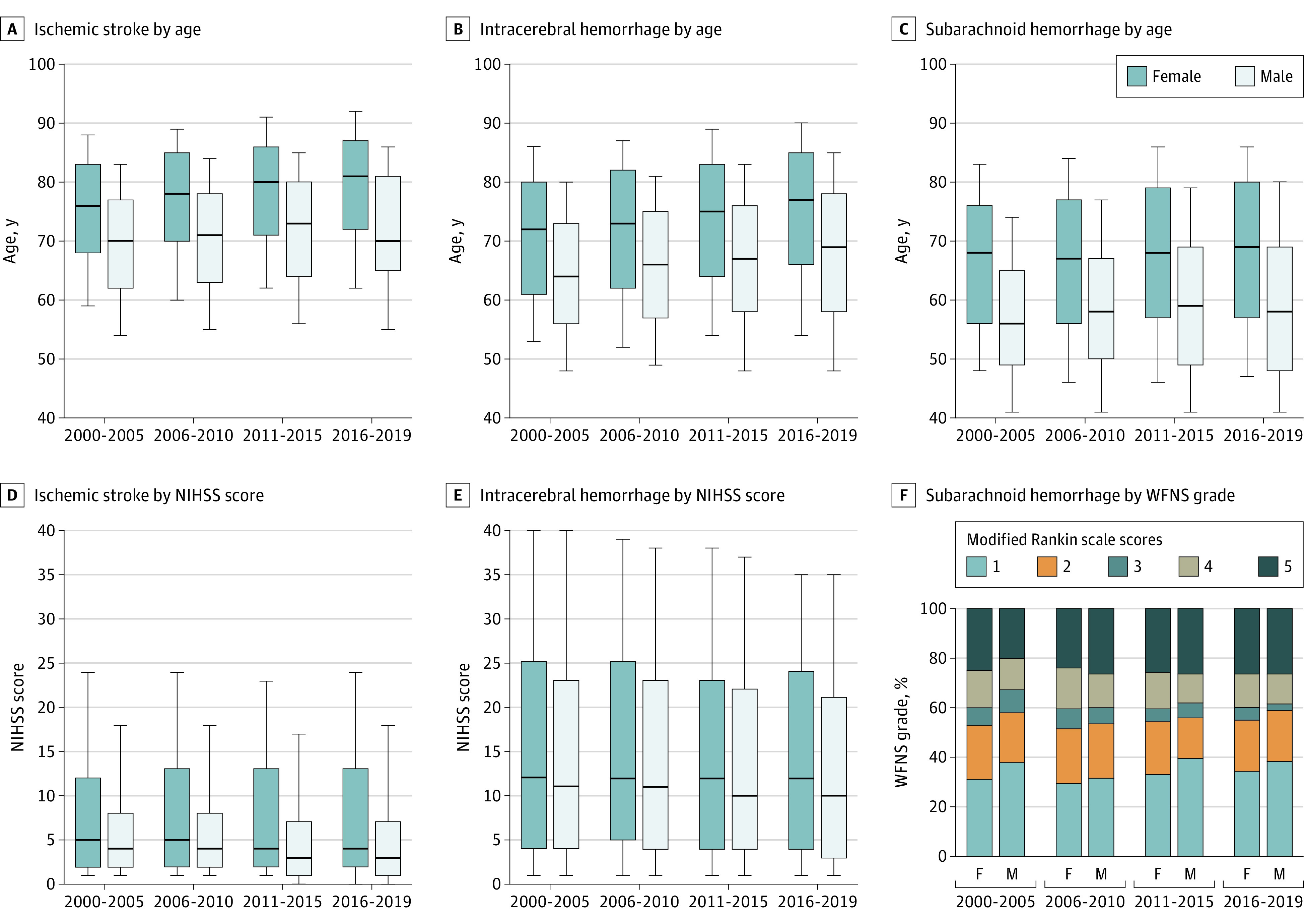
Age, National Institutes of Health Stroke Scale (NIHSS) Scores, and World Federation of Neurological Surgeons (WFNS) Grades at the Emergent Visit by Sex Boxes represent interquartile range, and lines across boxes indicate median values. Whiskers represent 10th percentile and 90th percentile values. All *P* values for sex differences in age are <.001. All *P* values for sex differences in NIHSS scores for ischemic stroke are <.001. *P* values for sex differences in NIHSS scores for intracerebral hemorrhage are .06 in 2000-2005, .006 in 2006-2010, .002 in 2011-2015, and <.001 in 2016-2019. *P* values for sex differences in WFNS grades are <.001 in 2000-2005, .81 in 2006-2010, 0.11 in 2011-2015, and .02 in 2016-2019. All *P* values for trends of age and NIHSS scores are <.001 in both sexes. *P* values for trends of WFNS grades are .28 for women and .64 for men.

The baseline characteristics and outcomes of patients are shown in Table 1. Of the 3 stroke types, patients with SAH were mostly female and were the youngest. Patients with ICH had higher NIHSS scores than those with IS. Discharge mRS scores were the highest in patients with ICH, and in-hospital mortality was the highest in those with SAH. Among the 3 major IS subtypes, patients with cardioembolism were mostly female, were the oldest, had the highest NIHSS and mRS scores, and had the highest mortality. History of stroke was more common in men than women with IS and SAH. Male patients with IS more often underwent acute reperfusion therapy than female patients. Initial NIHSS or WFNS scores and discharge mRS scores were higher in women than men for all stroke types. In-hospital mortality was higher in women than men with IS and lower in women with ICH. Figure 1A-C shows changes in the median age of patients by period. In all stroke types, women were older than men and ages at onset became older with time in both sexes.

**Table 1.** Baseline Characteristics and Outcomes of Patients

Variable	Total stroke	Ischemic stroke				Intracerebral hemorrhage	Subarachnoid hemorrhage
		Total	Cardioembolism	Large-artery atherosclerosis	Small-vessel occlusion		
No. of patients	183 082	135 268	38 896	42 302	37 541	36 014	11 800
Women, No. (%)	77 089 (42.1)	53 800 (39.8)	17 941 (46.1)	14 923 (35.3)	14 352 (38.2)	15 365 (42.7)	7924 (67.2)
Men, No. (%)	105 993 (57.9)	81 468 (60.2)	20 955 (53.9)	27 379 (64.7)	23 189 (61.8)	20 649 (57.3)	3876 (32.8)
Age, median (IQR), y							
Women	77 (67-84)	79 (70-85)	82 (75-88)	78 (70-85)	76 (67-83)	74 (63-83)	68 (56-78)
Men	70 (62-78)	72 (64-79)	75 (67-81)	72 (65-79)	69 (61-77)	67 (57-76)	57 (49-68)
* P* value^a^	<.001	<.001	<.001	<.001	<.001	<.001	<.001
NIHSS score, median (IQR)							
Women	NA	5 (2-13)	13 (4-22)	4 (2-10)	3 (1-5)	12 (4-24)	NA
Men	NA	3 (2-8)	7 (2-18)	4 (2-7)	3 (1-4)	11 (4-22)	NA
* P* value^a^	NA	<.001	<.001	<.001	<.001	<.001	NA
WFNS grade, median (IQR)							
Women	NA	NA	NA	NA	NA	NA	2 (1-5)
Men	NA	NA	NA	NA	NA	NA	2 (1-4)
* P* value^a^	NA	NA	NA	NA	NA	NA	<.001
History of stroke, No. (%)							
Women	17 393 (23.9)	13 114 (25.8)	4554 (27.3)	3808 (26.8)	3490 (25.5)	3509 (23.9)	770 (10.5)
Men	27 557 (27.5)	22 534 (29.2)	5639 (28.8)	8155 (31.2)	6545 (29.6)	4709 (23.9)	314 (8.8)
* P* value^a^	<.001	<.001	.002	<.001	<.001	.94	.007
Acute reperfusion therapy, No. (%)							
Women	NA	4629 (8.6)^b^	2983 (16.3)	758 (5.1)	271 (1.9)	NA	NA
Men	NA	7281 (8.9)^c^	3528 (16.8)	2301 (8.4)	472 (2.0)	NA	NA
* P* value^a^	NA	.03	.58	<.001	.32	NA	NA
Days of hospitalization, median (IQR)							
Women	21 (12-37)	18 (11-32)	22 (13-39)	20 (13-35)	15 (10-25)	24 (13-43)	31 (18-54)
Men	18 (11-32)	16 (10-29)	20 (12-35)	18 (12-32)	13 (9-21)	23 (11-41)	29 (17-51)
* P* value^a^	<.001	<.001	<.001	<.001	<.001	<.001	<.001
Discharge mRS score, median (IQR)							
Women	3 (1-5)	3 (1-4)	4 (2-5)	3 (1-4)	1 (1-3)	4 (2-5)	3 (0-5)
Men	2 (1-4)	2 (1-4)	3 (1-4)	2 (1-4)	1 (1-2)	4 (2-5)	2 (0-5)
* P* value^a^	<.001	<.001	<.001	<.001	<.001	<.001	<.001
In-hospital death, No. (%)							
Women	7007 (9.3)	3207 (6.1)	2273 (12.9)	528 (3.6)	90 (0.6)	2097 (13.9)	1703 (22.1)
Men	6709 (6.5)	2919 (3.6)	1688 (8.2)	746 (2.8)	135 (0.6)	3008 (14.9)	782 (20.9)
* P* value^a^	<.001	<.001	<.001	<.001	.59	.008	.13

Abbreviations: mRS, modified Rankin Score; NA, not applicable; NIHSS, National Institutes of Health Stroke Scale; WFNS, World Federation of Neurological Surgeons.

^a^ *P* value for sex differences. All *P* values for differences among 3 ischemic stroke subtypes (cardioembolism, large-artery atherosclerosis, and small-vessel occlusion) are <.001, except for history of stroke in women (*P* = .002). All *P* values for differences among 3 stroke types (ischemic stroke, intracerebral hemorrhage, and subarachnoid hemorrhage) are <.001.

^b^ 1.4% Between 2000 and 2005, 6.0% between 2006 and 2010, 8.8% between 2011 and 2015, and 17.3% between 2016 and 2019 (*P* < .05).

^c^ 2.1% Between 2000 and 2005, 7.4% between 2006 and 2010, 9.3% between 2011 and 2015, and 16.6% between 2016 and 2019 (*P* < .05).

NIHSS scores became lower throughout the 20 years regardless of sex in all stroke types and all IS subtypes, even adjusted by age and stroke history (eTable 2 in Supplement 1). Figure 1D-F shows a decrease in NIHSS scores in patients with IS and ICH over time. WFNS scores in patients with SAH did not show secular changes.

**Figure 2.**
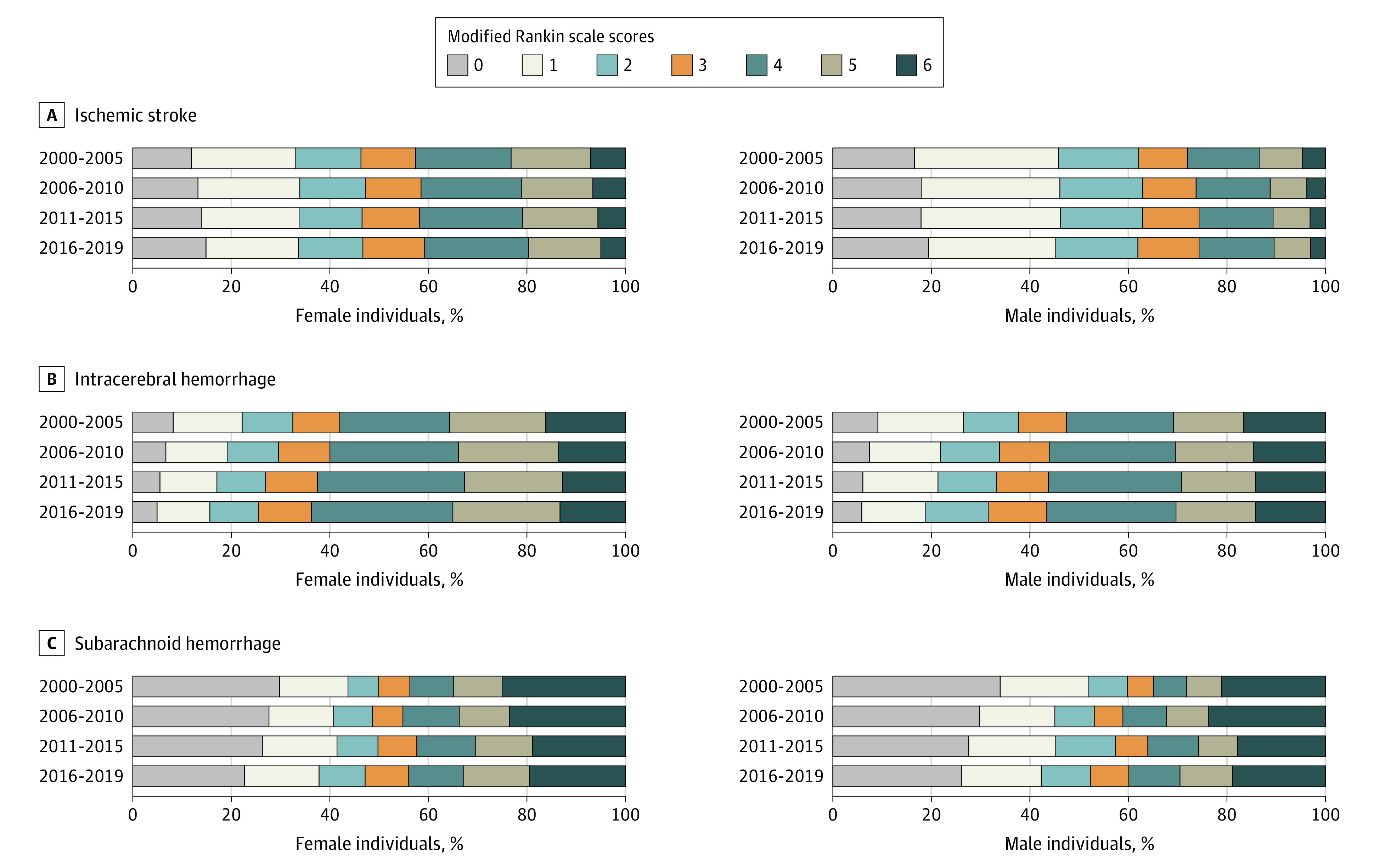
Modified Rankin Scale Scores at Discharge by Sex

In patients with IS, the proportion of favorable outcomes increased significantly after age adjustment (model 1) but no longer increased after further adjustment by NIHSS scores and stroke history (model 2) in women and men (Table 2 and Figure 2). The odds ratios of the proportion were lower after further adjustment by achievement of reperfusion therapy in both sexes (model 3), and the proportion decreased significantly in men. Both unfavorable outcomes and death decreased over time after any adjustments in both sexes (Table 3). Because 2005, 2010, and 2015 were years for appearance of new therapeutic technologies for IS in Japan as described above,^7^ changes in the outcomes by 5-year categories were also determined (eTable 3 in Supplement 1). The results were similar with those of per-year changes.

**Table 2.** Secular Changes in Favorable Outcomes at Discharge

Outcome	Odds ratio (95% CI)^a^			
	Crude	Model 1^b^	Model 2^c^	Model 3^d^
**Women**				
Total ischemic stroke	0.994 (0.995-1.003)	1.020 (1.015-1.024)	1.003 (0.998-1.009)	0.997 (0.991-1.003)
Cardioembolism	1.009 (1.002-1.017)	1.037 (1.029-1.045)	1.023 (1.012-1.034)	1.008 (0.997-1.019)
Large-artery atherosclerosis	1.010 (1.003-1.018)	1.028 (1.020-1.036)	1.004 (0.994-1.014)	1.002 (0.992-1.013)
Small-vessel occlusion	0.997 (0.989-1.005)	1.014 (1.005-1.022)	0.986 (0.975-0.997)	0.985 (0.974-0.995)
Intracerebral hemorrhage	0.984 (0.976-0.992)	0.994 (0.986-1.003)	0.980 (0.968-0.992)	NA
Subarachnoid hemorrhage	1.000 (0.990-1.010)	1.011 (1.000-1.022)	1.002 (0.989-1.016)	NA
**Men**				
Total ischemic stroke	1.002 (0.999-1.005)	1.015 (1.011-1.018)	0.995 (0.991-1.000)	0.990 (0.985-0.994)
Cardioembolism	1.006 (1.000-1.013)	1.023 (1.016-1.029)	1.007 (0.998-1.016)	0.993 (0.984-1.002)
Large-artery atherosclerosis	1.009 (1.003-1.015)	1.020 (1.014-1.026)	1.001 (0.993-1.008)	0.998 (0.991-1.006)
Small-vessel occlusion	0.997 (0.991-1.004)	1.009 (1.002-1.016)	0.982 (0.973-0.991)	0.980 (0.971-0.989)
Intracerebral hemorrhage	0.983 (0.976-0.990)	0.989 (0.982-0.996)	0.971 (0.961-0.982)	NA
Subarachnoid hemorrhage	0.996 (0.982-1.009)	1.002 (0.988-1.017)	0.989 (0.970-1.008)	NA

Abbreviations: NA, not applicable; NIHSS, National Institutes of Health Stroke Scale; WFNS, World Federation of Neurological Surgeons.

^a^ Odds ratio (95% CI) per 1 year.

^b^ Model 1 is adjusted by age.

^c^ Model 2 is adjusted by age, NIHSS score (WFNS grade for subarachnoid hemorrhage), and history of stroke.

^d^ Model 3 is adjusted by age, NIHSS score (WFNS grade for subarachnoid hemorrhage), history of stroke, and reperfusion therapy.

**Table 3.** Secular Changes in Poor Outcomes at Discharge

Outcome	Odds ratio (95% CI)^a^				
	Crude		Model 1^b^	Model 2^c^	Model 3^d^
Unfavorable outcome in women					
Ischemic stroke	0.989 (0.984-0.994)		0.967 (0.962-0.972)	0.974 (0.967-0.981)	0.981 (0.973-0.988)
Intracerebral hemorrhage	0.996 (0.988-1.003)		0.984 (0.976-0.992)	0.987 (0.974-0.999)	NA
Subarachnoid hemorrhage	0.986 (0.975-0.996)		0.974 (0.963-0.985)	0.973 (0.959-0.987)	NA
Unfavorable outcome in men					
Ischemic stroke	0.982 (0.977-0.987)		0.967 (0.962-0.972)	0.978 (0.971-0.985)	0.984 (0.977-0.991)
Intracerebral hemorrhage	0.999 (0.993-1.007)		0.991 (0.985-0.998)	1.001 (0.990-1.013)	NA
Subarachnoid hemorrhage	0.989 (0.974-1.004)		0.981(0.966-0.997)	0.976 (0.956-0.995)	NA
In-hospital death in women					
Ischemic stroke	0.975 (0.967-0.983)		0.959 (0.951-0.967)	0.969 (0.959-0.979)	0.967 (0.957-0.978)
Intracerebral hemorrhage	0.984 (0.974-0.994)		0.976 (0.965-0.986)	0.978 (0.963-0.994)	NA
Subarachnoid hemorrhage	0.970 (0.958-0.982)		0.960 (0.948-0.972)	0.951 (0.937-0.965)	NA
In-hospital death in men					
Ischemic stroke	0.964 (0.956-0.972)		0.950 (0.942-0.958)	0.966 (0.956-0.976)	0.967 (0.956-0.977)
Intracerebral hemorrhage	0.992 (0.983-1.001)		0.985 (0.977-0.994)	0.998 (0.985-1.012)	NA
Subarachnoid hemorrhage	0.979 (0.962-0.996)		0.972 (0.955-0.990)	0.965 (0.945-0.986)	NA

Abbreviations: NA, not applicable; NIHSS, National Institutes of Health Stroke Scale; WFNS, World Federation of Neurological Surgeons.

^a^ Odds ratio (95% CI) per 1 year.

^b^ Model 1 is adjusted by age.

^c^ Model 2 is adjusted by age, NIHSS score (WFNS grade for subarachnoid hemorrhage), and history of stroke.

^d^ Model 3 is adjusted by age, NIHSS score (WFNS grade for subarachnoid hemorrhage), history of stroke, and reperfusion therapy.

In both patients with cardioembolism and those with large-artery atherosclerosis, the proportion of favorable outcomes increased after model 1 adjustment, remained increased only in female patients with cardioembolic IS after model 2 adjustment, and no longer increased in either sex after model 3 adjustment (Table 2). In these 2 IS subtypes, unfavorable outcomes and death became less common after any adjustments in both sexes (eTable 4 in Supplement 1).

In patients with IS with small vessel occlusion, the proportion of favorable outcomes increased after model 1 adjustment and decreased after model 2 and model 3 adjustments in both sexes (Table 2). Neither the proportions of unfavorable outcomes nor the proportions of deaths showed secular changes after any adjustments except for model 1 adjustment for unfavorable outcomes in women (eTable 4 in Supplement 1).

In patients with ICH, favorable outcomes became less common after both age adjustment (model 1) and further adjustment by NIHSS scores and stroke history (model 2) in both sexes, except for model 1 adjustment in women (Table 2 and Figure 2). Both unfavorable outcomes and deaths became less common after both model 1 and model 2 adjustment in women and did not show secular change after model 2 adjustment in men (Table 3).

In patients with SAH, favorable outcomes did not show secular change in proportions, and both unfavorable outcomes and deaths became less common after both age adjustment (model 1) and further adjustment by WFNS scores and stroke history (model 2) in both sexes.

## Discussion

During the past 20 years up to 2019, the JSDB study enrolled more than 200 000 patients with acute strokes or transient ischemic attacks from stroke centers distributed evenly throughout Japan. In the present analysis, there were several major new findings. First, median ages at onset increased, and the NIHSS/WFNS scores decreased on multivariable adjustment in all stroke types. Second, functional outcomes at hospital discharge improved in patients with IS with time, but the improvement was no longer clear on multivariable adjustment including reperfusion therapy. Third, the proportion of favorable outcomes (unfavorable outcome, in-hospital death) in hemorrhagic strokes decreased or was unchanged but never increased throughout the 20 years. Sex differences in 20-year trends of each outcome were generally inconspicuous. To our knowledge, this was the first study to examine changes in functional outcomes after ischemic and hemorrhagic strokes over 2 decades using quantitative scales for a large population.

In the present cohort, NIHSS scores decreased over time in both sexes in all 3 IS subtypes and ICH, regardless of adjustment for age and stroke history. The trends were similar to the above results from the Austrian and Israeli registries.^9,10^ The decrease could be explained by the 2 reasons proposed previouly^10^: improvement in preventive therapy and changes in case mix. To give typical examples of the first reason, strict blood pressure control enabled by recent strong antihypertensives and clarification of the importance of a low-salt diet could decrease the onset of large ICH,^32^ and the diffusion of anticoagulation for patients with atrial fibrillation by the widespread use of direct oral anticoagulants could prevent huge infarcts due to large cardiac thrombi. In fact, the impact on the decrease of coefficients was largest in female patients with cardioembolism. The second reason, changes in case mix, means, for example, development of brain imaging modalities such as diffusion-weighted imaging that accurately differentiates minor stroke from mimics and would increase registration of such minor stroke.

Representative trends in functional outcomes after IS can be seen in patients with nonlacunar stroke in the present study. Favorable outcomes increased over time after age adjustment, and the odds ratio gradually decreased after NIHSS score adjustment and after further adjustment by reperfusion therapy, as a representative decisive therapy.^33,34^ In male patients with IS, the proportion of favorable outcomes finally decreased after adjustment by reperfusion therapy. Thus, reperfusion therapy seemed to be a reason for the gradual improvement of functional outcomes over 20 years. Decreased proportions of poor outcomes were also clear in patients with nonlacunar subtypes even after NIHSS score adjustment. Widespread use of dual antiplatelet therapy,^35^ development of early rehabilitation,^36^ and early initiation of anticoagulation after stroke onset using direct oral anticoagulants might also improve functional outcomes.^37^ In contrast, trends in favorable and poor outcomes were somewhat unclear in patients with lacunar stroke, suggesting that lacunar stroke received the least benefits from the recent development of acute therapies among the 3 subtypes, especially mechanical thrombectomy, owing to the absence of target large arteries.

In female and male patients with ICH, favorable outcomes became less common, and poor outcomes showed sex differences in trends on multivariate analysis. The tendency for improvement of functional outcome seemed unclear compared with patients with IS. This partly suggested the lack of powerful acute therapies equal to reperfusion therapy for IS, although intensive blood pressure lowering and hemostatic agents specific to each anticoagulant are regarded as promising therapies.^31^ Patients with SAH also showed an inconspicuous tendency for improvement of outcome. A recent increase in antithrombotics-associated ICH, also shown in the JSDB population (data not shown), might also affect the initial severity and functional outcome.^38,39^

There are evident differences in several stroke features between women and men.^28,29,30^ In the present study, women constituted 42.1% of overall stroke patients, similar to the percentage with the hospital-based registries from China (38.4%),^19^ Korea (42.3%),^12^ and Israel (43.0%).^10^ Age-specific stroke incidence was lower for women than men at ages 55 to 75 years and similar at the other ages in the 2016 Global Burden of Diseases, Injuries, and Risk Factors Study.^40^ At least in East Asia, the ratio of female inpatients with stroke to their male counterparts would be approximately 2:3. Women were around 7 or more years older than men at stroke onset in any stroke types and any IS subtypes, and ages at onset became higher with time in both sexes. Women had higher admission NIHSS/WFNS scores than men in any stroke type and any IS subtype on crude analysis. However, sex differences of 20-year trends in functional outcomes were generally unclear in any stroke types, presumably partly because Japanese women received relatively equally the benefits of medical care during acute hospitalization to Japanese men. Although limited access to medical resources has been an essential limitation for stroke in women,^29^ there were only modest sex-related differences in the achievement of reperfusion therapy in the present study; women underwent reperfusion therapy somewhat more frequently between 2016 and 2020 (17.3% vs 16.6%; Table 1).

### Strengths and Limitations

The strengths of the present study were its duration and the population size of the database. The JSDB study belongs to the oldest group that commenced patient registration around 2000 among 28 national registries listed in a systematic review.^8^ Registration of approximately 10 000 patients per year enabled us to analyze a substantial number of patients by year unit. Web-based data accumulation from the beginning with thorough cleansing has enabled accurate and well-preserved data.

The limitations of the JSDB study include, first, the ongoing operation with secular changes in the participating sites, as several sites joined and a smaller number of sites became inactive midway. Second, the JSDB study registered only approximately 3% (recently, approximately 6%) of patients with stroke throughout Japan based on the estimated number of total domestic patients with stroke by regional population-based surveys. Because high-volume stroke centers tended to join the JSDB study, the present results would not be generalizable to low-volume hospitals in Japan. Third, 6388 patients (3.2%) were excluded owing to unavailable data on stroke types or demography. These patients had the lower median NIHSS scores and lower percentage of favorable outcome and in-hospital mortality than the 183 082 included patients. Unfavorable outcome was similarly common. Two-thirds of the excluded patients belonged to the third category of years (2011-2015), and the proportion of such excluded patients was 1.0% (465 of 45 849) in the latest category (2016-2019). Fourth, concepts of some of the collected data varied according to the currents of times over the 20-year period. For example, the diagnosis of IS with other determined and undetermined causes varied according to development of diagnostic technology and trends of theories, such as reevaluation of cryptogenic stroke as embolic stroke with undetermined sources.^41^ The present study did not focus on such inconsistent items. Fifth, functional outcomes were assessed at the time of discharge from the acute hospital around a median of 20 days after stroke onset because longer-term outcomes were not collected for all patients. Finally, data from 2020 were not included, and the effect of the COVID-19 pandemic was therefore not assessed.

## Conclusions

In conclusion, stroke became milder in severity during the past 20 years regardless of sex or stroke types although age at stroke onset became older in the nationwide stroke registry in Japan. Short-term functional outcomes at hospital discharge improved gradually in patients with IS, presumably partly owing to development of acute reperfusion therapy. In contrast, outcomes of patients with hemorrhagic stroke did not clearly improve during the same period. Such differences among stroke types might reflect the existence of decisively effective acute therapeutic strategies for IS and not for ICH and SAH.

## Notes

Supplement 1. eFigure. Flow diagram eTable 1. List of the Japan Stroke Data Bank Investigators eTable 2. Secular changes in National Institutes of Health Stroke Scale scores at the emergent visit eTable 3. Secular changes in ischemic stroke outcomes at discharge according to four categories of years eTable 4. Secular changes in poor outcomes at discharge by ischemic stroke subtypes

Supplement 2. Nonauthor Collaborators. Japan Stroke Data Bank Investigators
