# Supplementary material for: Twenty-Year Change in Severity and Outcome of Ischemic and Hemorrhagic Strokes
Source: JAMA Neurol. 2021 Dec 6;79(1):1–9. doi: 10.1001/jamaneurol.2021.4346 (PMC8649912; doi:10.1001/jamaneurol.2021.4346)
Supplement: Supplement 1. — eFigure. Flow diagram eTable 1. List of the Japan Stroke Data Bank Investigators eTable 2. Secular changes in National Institutes of Health Stroke Scale scores at the emergent visit eTable 3. Secular changes in ischemic stroke outcomes at discharge according to four categories of years eTable 4. Secular changes in poor outcomes at discharge by ischemic stroke subtypes [file jamaneurol-e214346-s001.pdf]

Supplemental Online Content

Toyoda K, Yoshimura S, Nakai M, et al; Japan Stroke Data Bank Investigators. Twenty-year change in severity and outcome of ischemic and hemorrhagic strokes. *JAMA Neurol*. Published online December 6, 2021. doi:10.1001/jamaneurol.2021.4346

eFigure. Flow diagram
eTable 1. List of the Japan Stroke Data Bank Investigators
eTable 2. Secular changes in National Institutes of Health Stroke Scale scores at the emergent visit
eTable 3. Secular changes in ischemic stroke outcomes at discharge according to four categories of years
eTable 4. Secular changes in poor outcomes at discharge by ischemic stroke subtypes

This supplemental material has been provided by the authors to give readers additional information about their work.

© 2021 Toyoda K et al. *JAMA Neurology.*

eFigure. Flow diagram

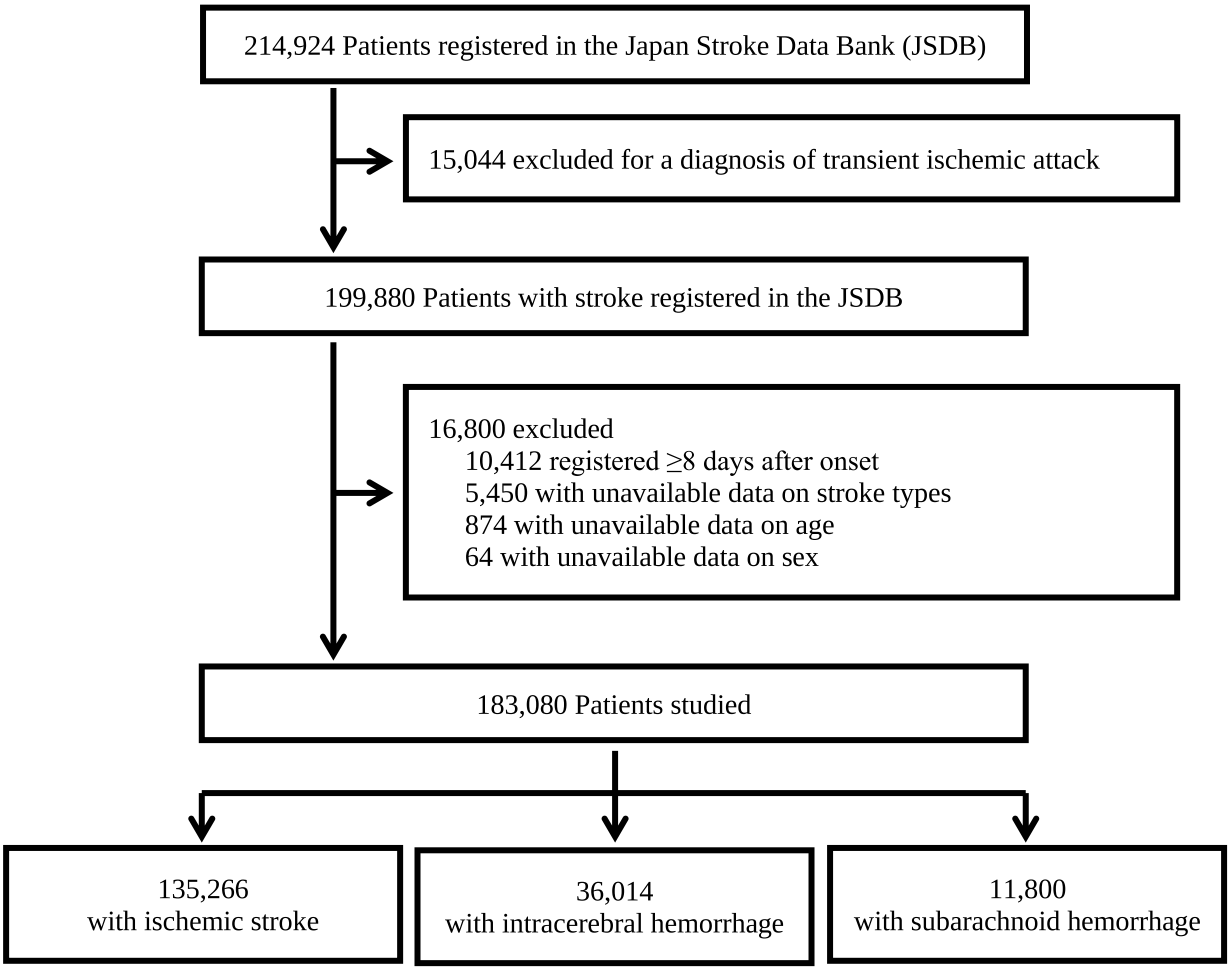

© 2021 Toyoda K et al. *JAMA Neurology.*

eTable 1. List of the Japan Stroke Data Bank Investigators

Adviser	Shotai Kobayashi, MD; Kazuo Minematsu, MD
Steering committee	Koji Iihara, MD; Ryo Itabashi, MD; Kenji Kamiyama, MD; Takanari Kitazono, MD; Masatoshi Koga, MD; Yasuhiro Miyamoto, MD; Kuniaki Ogasawara, MD; Shigeru Nogawa, MD; Kazunori Toyoda, MD (Chair); Masaaki Uno, MD; Fusao Igawa, MD; Shuhei Yamaguchi, MD
Central office in the National Cerebral and Cardiovascular Center	Sohei Yoshimura, MD; Kaori Miwa, MD; Junpei Koge, MD; Akiko Ishigami, MD; Ai Ito (Department of Cerebrovascular Medicine)<br><br>Yoshitaka Iwanaga, MD; Michikazu Nakai, PhD; Yusuke Sasahara, PhD; Shinichi Wada, MD (Center for Cerebral and Cardiovascular Disease Information)

© 2021 Toyoda K et al. *JAMA Neurology.*

eTable 2. Secular changes in National Institutes of Health Stroke Scale scores at the emergent visit

	Women		Men	
	**Crude**	**Adjusted***	**Crude**	**Adjusted***
**Total ischemic stroke**	-0.050 (-0.071 – -0.029)	-0.084 (-0.105 – -0.064)	-0.073 (-0.087 – -0.059)	-0.088 (-0.102 – -0.074)
**Cardioembolism**	-0.105 (-0.146 – -0.063)	-0.151 (-0.192 – -0.109)	-0.083 (-0.120 – -0.047)	-0.107 (-0.144 – -0.071)
**Large-artery atherosclerosis**	-0.118 (-0.151 – -0.084)	-0.135 (-0.168 – -0.102)	-0.100 (-0.121 – -0.079)	-0.107 (-0.127 – -0.086)
**Small-vessel occlusion**	-0.063 (-0.082 – -0.045)	-0.069 (-0.086 – -0.051)	-0.062 (-0.074 – -0.050)	-0.066 (-0.077 – -0.054)
**Intracerebral hemorrhage**	-0.087 (-0.140 – 0.033)	-0.109 (-0.162 – -0.055)	-0.082 (-0.128 – -0.036)	-0.082 (-0.128 – -0.036)

Coefficient (95% confidence index)

* adjusted by age and history of stroke

© 2021 Toyoda K et al. *JAMA Neurology.*

eTable 3. Secular changes in ischemic stroke outcomes at discharge according to four categories of years

	Crude	Model 1	Model 2	Model 3
**Women**				
Favorable outcome	1.004 (0.984 –1.024)	1.098 (1.075 –1.122)	1.017 (0.989 –1.046)	0.987 (0.959 –1.016)
Unfavorable outcome	0.945 (0.922 –0.969)	0.855 (0.833 –0.878)	0.882 (0.850 –0.915)	0.911 (0.877 –0.946)
In-hospital death	0.887 (0.851 –0.923)	0.823 (0.790 –0.858)	0.856 (0.813 –0.900)	0.850 (0.807 –0.896)
**Men**				
Favorable outcome	1.004 (0.987 –1.021)	1.062 (1.044 –1.081)	0.974 (0.952 –0.997)	0.946 (0.924 –0.968)
Unfavorable outcome	0.918 (0.895 –0.942)	0.856 (0.834 –0.879)	0.887 (0.856 –0.920)	0.915 (0.882 –0.949)
In-hospital death	0.845 (0.810 –0.882)	0.793 (0.759 –0.828)	0.844 (0.802 –0.889)	0.849 (0.805 –0.895)

Odds ratio (95% confidence interval) per 1- category

Four categories of years are 2000-2005, 2006-2010, 2011-2015, 2016-2019

Model 1: adjusted by age

Model 2: adjusted by age, NIHSS score (WFNS grade for subarachnoid hemorrhage), and history of stroke

Model 3: adjusted by age, NIHSS score (WFNS grade for subarachnoid hemorrhage), history of stroke, and reperfusion therapy

© 2021 Toyoda K et al. *JAMA Neurology.*

eTable 4. Secular changes in poor outcomes at discharge by ischemic stroke subtypes

	Crude	Model 1	Model 2	Model 3
**Unfavorable outcome in women**				
Cardioembolism	0.978 (0.971 – 0.985)	0.956 (0.949 – 0.963)	0.963 (0.953 – 0.973)	0.977 (0.966 – 0.987)
Large-artery atherosclerosis	0.970 (0.961 – 0.980)	0.952 (0.943 – 0.962)	0.970 (0.956 – 0.983)	0.969 (0.955 – 0.983)
Small-vessel occlusion	1.000 (0.984 – 1.017)	0.979 (0.962 – 0.995)	0.993 (0.971 – 1.015)	0.993 (0.971 – 1.015)
**Unfavorable outcome in men**				
Cardioembolism	0.976 (0.969 – 0.983)	0.959 (0.952 – 0.967)	0.964(0.954 – 0.974)	0.978 (0.967 – 0.989)
Large-artery atherosclerosis	0.968 (0.959 – 0.976)	0.956 (0.947 – 0.964)	0.974 (0.963 – 0.986)	0.975 (0.963 – 0.986)
Small-vessel occlusion	1.013 (0.996 – 1.031)	0.996 (0.979 – 1.014)	1.011 (0.989 – 1.034)	1.012 (0.989 – 1.035)
**In-hospital death in women**				
Cardioembolism	0.970 (0.961 – 0.980)	0.958 (0.948 – 0.967)	0.969 (0.957 – 0.981)	0.973 (0.960 – 0.985)
Large-artery atherosclerosis	0.925 (0.907 – 0.944)	0.910 (0.892 – 0.929)	0.933 (0.912– 0.955)	0.931 (0.909 – 0.953)
Small-vessel occlusion	1.007 (0.967 – 1.049)	0.988 (0.947 – 1.030)	0.988 (0.941 – 1.038)	0.988 (0.941 – 1.039)
**In-hospital death in men**				
Cardioembolism	0.968 (0.957 – 0.979)	0.954 (0.943 – 0.965)	0.966 (0.953 – 0.979)	0.974 (0.960 – 0.988)
Large-artery atherosclerosis	0.930 (0.914 – 0.945)	0.919 (0.904 – 0.935)	0.948 (0.930 – 0.967)	0.942 (0.924 – 0.961)
Small-vessel occlusion	0.999 (0.964 – 1.035)	0.976 (0.942 – 1.012)	1.000 (0.958 – 1.043)	1.000 (0.958 – 1.043)

Odds ratio (95% confidence interval) per 1-year

Model 1: adjusted by age

Model 2: adjusted by age, NIHSS score (WFNS grade for subarachnoid hemorrhage), and history of stroke

Model 3: adjusted by age, NIHSS score (WFNS grade for subarachnoid hemorrhage), history of stroke, and reperfusion therapy

© 2021 Toyoda K et al. *JAMA Neurology.*
