# Supplementary material for: Twenty-Year Change in Severity and Outcome of Ischemic and Hemorrhagic Strokes
Source: JAMA Neurol. 2021 Dec 6;79(1):1–9. doi: 10.1001/jamaneurol.2021.4346 (PMC8649912; doi:10.1001/jamaneurol.2021.4346)
Supplement: Supplement 2. — Nonauthor Collaborators. Japan Stroke Data Bank Investigators [file jamaneurol-e214346-s002.pdf]

Supplemental Online Content: Nonauthor Collaborators

*Indicates required information. Only first name, last name, and suffix will appear in PubMed.

*Group Name(s): Japan Stroke Data Bank Investigators							
***First Name and Middle Initial(s)**	***Last Name**	***Suffix (eg, Jr, III)**	Academic Degrees	Institution	Location (city, state/province, country)	Role or Contribution, eg, chair, principal investigator	Group (if more than 1 Group listed in the byline) and/or Subgroup (eg, Steering Committee)
Koji	Iihara		MD, PhD	National Cerebral and Cardiovascular Center	Suita, Osaka, Japan	Steering Committee member	
Ryo	Itabashi		MD, PhD	Iwate Medical University	Yahaba, Iwate, Japan	Steering Committee member	
Takanari	Kitazono		MD, PhD	Kyushu University	Fukuoka, Japan	Steering Committee member	
Kuniaki	Ogasawara		MD, PhD	Iwate Medical University	Yahaba, Iwate, Japan	Steering Committee member	
Shigeru	Nogawa		MD, PhD	Tokai University Hachioji Hospital	Hachioji, Tokyo, Japan	Steering Committee member	
Masaaki	Uno		MD, PhD	Kawasaki Medical School	Kurashiki, Okayama, Japan	Steering Committee member	
Fusao	Ikawa		MD, PhD	Shimane Prefectural Central Hospital	Izumo, Shimane, Japan	Steering Committee member	
Shuhei	Yamaguchi		MD, PhD	Shimane Prefectural Central Hospital	Izumo, Shimane, Japan	Steering Committee member	
Ai	Ito		-	National Cerebral and Cardiovascular Center	Suita, Osaka, Japan	Secretary	
